# Transposition of Teeth

**Published:** 1846-09

**Authors:** 


*Transposition of Teeth.*—Dr. Noyes recently presented us with a left lateral incisor which he had just extracted from between the central incisors of a girl about fourteen years of age. Aberrations of this sort in the growth and eruption of the teeth, though of very rare occurrence, are, nevertheless, occasionally met with. Two similar examples have fallen under our own observation—one consisted in the transposition of a cuspidatus and bicuspis; the other in that of a cuspidatus and lateral incisor. In the case mentioned to us by Dr. N., the extraction of the wrongly placed lateral incisor, which was largely developed, was rendered necessary to make room for the other three incisors between the cuspidati, which, by its presence, were partially crowded from the arch.—*Balt. Ed.*

